# STELLAR‐CB: Synthetic Temporal LSTM for Livestock Activity Recognition—Cow Behaviour

**DOI:** 10.1002/vms3.70827

**Published:** 2026-06-01

**Authors:** Ghufran Ahmed, Rauf Ahmed Shams Malick, Ahmad Sami Al‐Shamayleh, Muhammad Adnan Ayub, Usama Antuley, Twaha Ahmed Minai, Muhammad Faisal Khan, Muhammad Faraz Hyder, Muhammad Mubashir Khan, Adnan Akhunzada

**Affiliations:** ^1^ Department of Computer Science School of Computing National University of Computer and Emerging Sciences Karachi Pakistan; ^2^ Department of Computer Science GU Tech Al Ghazali University Karachi Pakistan; ^3^ Department of Data Science and Artificial Intelligence Faculty of Information Technology Al‐Ahliyya Amman University Amman Jordan; ^4^ Dawood University of Engineering & Technology Karachi Pakistan; ^5^ Department of CS & IT NED University of Engineering & Technology Karachi Pakistan; ^6^ College of Computing and Information Technology University of Doha for Science & Technology Doha Qatar

**Keywords:** cow behaviour classification, data augmentation, deep learning, livestock activity recognition, long‐short‐term memory (LSTM) networks, precision livestock farming (PLF), synthetic minority oversampling technique (SMOTE), time series data analysis

## Abstract

Precision livestock farming (PLF) leverages activity sensors to monitor behaviours like grazing, resting and walking, yet class imbalance in datasets often leads to underrepresentation of minority behaviours such as ‘escaping’ and ‘being mounted.’ This study proposes a novel framework combining long short‐term memory (LSTM) networks with the synthetic minority oversampling technique (SMOTE) to address this challenge. Unlike existing methods that use complex SMOTE variants such as DeepSMOTE or latent space augmentations, which add computational complexity and overhead, our approach integrates simple SMOTE with non‐overlapping windowed segmentation, preserving sequential patterns during synthetic data generation while augmenting minority classes. The LSTM architecture captures temporal dependencies in the balanced dataset, enabling robust behaviour recognition. Evaluated on a composite accelerometer dataset derived from three distinct cows, the framework generalises across breeds, overcoming limitations of breed‐specific models. It achieves state‐of‐the‐art performance with 97.24% accuracy, 97.56% precision, 97.24% recall and a 97.29% F1‐score, significantly improving detection of rare behaviours without compromising majority class precision. By unifying data from multiple cows, the model ensures robustness to behavioural variability, enhancing scalability for diverse farming environments. The simplicity of using basic SMOTE reduces computational overhead, making the solution practical for real‐world deployment. This work bridges classical data balancing techniques with modern deep learning, offering a resource‐efficient blueprint for handling imbalanced time‐series data in agricultural AI. The results advance precision livestock farming by improving the reliability of automated behaviour monitoring, directly contributing to enhanced animal welfare and farm productivity through accessible, breed‐agnostic AI tools.

## Graphical abstract

This study introduces a novel framework combining LSTM networks with SMOTE to address class imbalance in precision livestock farming. It improves the detection of rare behaviours in livestock, achieving state‐of‐the‐art performance while reducing computational overhead, offering a practical, breed‐agnostic solution for enhancing automated behaviour monitoring and farm productivity.

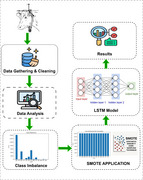

## 1 Introduction

Precision livestock farming (PLF) is a revolutionary livestock production method that employs high‐tech instruments to evaluate various parameters regarding the behaviour, health and well‐being of animals (Berckmans 2017). Among the technologies implemented, wearable sensors, principally accelerometers, are significant devices conducive to the live tracking of animal behavioural activities (Roqueto dos Reis 2023), (Barasa 2021). Use of accelerometers ushers the identification of subtle movement signals that indicate grazing, walking, lying, as well as rumination, ultimately allowing the automatic recognition of these behaviours. Such observations are significant in enhancing the production methodologies, improving the quality of animal living, as well as sustainable production.

Yet numerous problems occur as machine learning methods are utilised to process accelerometer data (Balasso et al. 2021). The first problem concerns the inherent behaviour dataset imbalance. Although behaviour such as resting prevails over other behaviour such as grazing or walking, the imbalance adversely affects the common classifier's performance, creating skewed prediction outcomes, ultimately nullifying the validity of the automatic behaviour measurement. Moreover, the vast bulk of the existing research employs naïve learning models or traditional statistical processes that do not learn the rich, complex temporal relationships embedded within sequential accelerometer data.

In addressing these challenges, researchers have increasingly turned to deep learning architectures, particularly recurrent neural networks (RNNs) and their more advanced forms, long short‐term memory networks (LSTMs), which are purpose‐built for managing time series data (Russel and Selvaraj 2024, Surana and Sharma 2024, Gao et al. 2023, Khin et al. 2024, Kumar et al. 2023). LSTMs excel in recognising long‐term dependencies and complex patterns, which makes them suitable for tasks that involve behaviour classification. However, this field is hindered by the significant problem of unbalanced datasets that compromise the performance of these models.

This research is inspired by earlier research works and bridges those gaps by creating a new framework employing LSTM along with the synthetic minority over‐sampling technique to deal with dataset imbalance applied to non‐overlapping, behaviour‐homogeneous 5‐s windows to preserve short‐horizon sequential structure (Blagus and Lusa 2013, Pradipta et al. 2021). In this paper, an experimental dataset has been derived from wearable accelerometers attached to cows, which has become a benchmark to evaluate the efficiency of machine learning models in recent studies. The proposed framework advances the state of cow behaviour classification and introduces the latest development in SMOTE to enhance the performance of machine learning models in agricultural applications.

The present investigation is guided by the seminal work of Fernández et al. (2018), where the fundamental mechanism of SMOTE is explained, which creates synthetic samples through the interpolation of instances from the minority class. This mechanism proved particularly efficient to achieve balance within largely imbalanced data sets, that is a common issue within the agricultural domain and animal behaviour general classification. Beyond that, the versatility of SMOTE reaches across numerous fields. Hence, this research study establishes the grounds of its use within cattle behaviour analysis, whereby data sets comprise predominantly class imbalances, for example, separating abnormal and normal behaviour patterns. This research ensures minority behaviour classes remain well‐specified, owing to their limited representation within the data set and will enhance the accuracy of the classifier.

Furthermore, the current study incorporates advanced variants of SMOTE based on the findings of Matharaarachchi et al. (2024) to minimise the impact of outliers and outlying instances on the cow behaviour dataset. The application of distance extSMOTE and BGMM SMOTE reduces the impact of outliers, a common phenomenon in real‐world agriculture datasets due to the inherent variability in the behaviour of animals. The advanced SMOTE methodologies produce more illustrative synthetic instances by considering the intricate and rare, high‐variance behaviours such as escaping, attacking and being mounted on the dataset, thereby augmenting the resilience and generalisation proficiency of the LSTM model. The capacity to adequately address outliers and atypical cases guarantees that the model is equipped to proficiently accommodate the varied spectrum of behaviours displayed by cows, an essential aspect for precise classification.

Furthermore, applying SMOTE along with deep learning done by Dablain et al. (2023) through their DeepSMOTE technique is similar to the LSTM‐based framework proposed in this paper. DeepSMOTE creates synthetic samples in the latent space and reconstructs them in the original data space, thereby enhancing the diversity of the minority class while maintaining the important features of the data. This method proved to be of particular utility in dealing with highly dimensional and complex datasets such as those created with the help of wearable accelerometers. The application of deep learning methodology coupled with SMOTE has allowed this study to effectively address the issue of class imbalance and consequently detect detailed patterns in the time‐sequencing and behavioural dimensions important for cattle precise classification.

According to Kashongwe et al. (2024) in their study on prediction of mastitis in dairy cows, class imbalance is a key challenge in predicting cow behaviour. It follows that SMOTE‐based balancing of class distributions and enhancing prediction accuracy will form a good basis for the use of SMOTE in livestock health monitoring.

Similarly, in the context of classifying animal behaviour, the combination of SMOTE with the LSTM‐based design allows minority behaviour that might go undetected without it to be captured as well as causing biasness in learning, thus boosting both recall and general classification accuracy. The ability of SMOTE to balance datasets in agricultural research thus resonates with its applied relevance and ability to enhance the correctness of cattle behaviour classifications. The potential that SMOTE holds to the pursue of data augmentation that is strong, combined with the deep learning design of LSTM, will render this a new and very efficient way through which the model handles class imbalances, outliers and complex data structures to classify cow behaviour. The methodology thus proposed will achieve significant improvements to minority classes classification accuracy while maintaining the superb majority classes performance. The recent developments confirm the use of behaviour data through wearable sensors not only to perceive activity and animal welfare but also to contribute to reproductive control and decision‐making on the farm (Cavallini et al. 2025, Lamanna et al. 2025)

The contributions of this work are as follows.

1. **Introduction of SMOTE for Dataset Balancing**: We apply SMOTE to generate synthetic samples for under‐represented behaviours, thus mitigating the effects of imbalance and improving overall model performance.
2. **Enhanced behaviour Classification Using LSTMs**: Leveraging the sequential capabilities of LSTMs, we achieve state‐of‐the‐art accuracy in cow behaviour classification.
3. **Comprehensive Evaluation**: The proposed framework is rigorously evaluated on a standard cow behaviour dataset, demonstrating substantial improvements over existing methods.

This study advances the field of precision livestock farming and provides a practical framework to address similar challenges in other domains that involve sequential data and class imbalance. The findings contribute to developing efficient, accurate and scalable solutions for animal behaviour monitoring, ultimately promoting better livestock management practices and animal welfare.

The rest of the paper is outlined as follows: Section 2 contains a detailed overview of previous studies in precision livestock farming, where sensor‐driven behaviour analysis and machine learning‐based techniques are put into perspective. Section 3 is devoted to the characteristics of the dataset used, the configuration used for the work is described and important features are extracted. Section 4 outlines the proposed method of pre‐processing the data, SMOTE augmentation and an LSTM‐based classification model. The results are given in Section 5, followed by a comparison with the existing literature in terms of various standard classification metrics, together with their associated performances. Section 6 is about the limitations and future work of this study. Finally, Section 7 concludes the study by summarising the main findings and contributions, which include the impact of SMOTE and deep learning on advances in classifying livestock behaviour.

## 2 Related Work

Precision livestock farming (PLF) is an area where there has been significant advancement, particularly by applying machine learning techniques to the study of cow behaviour from sensor data. The following are some of the significant previous research works that are closest to our work in methodologies, datasets and challenges.

### 2.1 Cow Behaviour Monitoring Using Accelerometer Data

A number of studies have employed accelerometer data for the classification of cow behaviour. For example, Russel and Selvaraj (Russel and Selvaraj 2024) introduced a deep learning model that employs accelerometer data for behaviour classification such as grazing, lying and walking with the best accuracy rate of 98. 7% in three datasets. Their method used convolutional layers, batch normalisation and rectified linear unit (ReLU) activation functions to extract the movement signature of the cows in a novel manner, thus greatly enhancing automated livestock management.

El Moutaouakil and Falih (2024) applied RNNs to sequential analysis of accelerometer signals to classify rest, movement, rumination and feeding behaviours. The experiment demonstrated the ability of RNN in representing temporal dependency and showed very high recall and precision in behaviour classification, thus providing further evidence towards the use of sequence‐based models such as LSTMs for time‐series data.

Bartels et al. (2022) extended this work by proposing TinyCowNet, a lightweight RNN framework designed specifically for memory‐ and power‐constrained edge devices. By focusing on cow behaviour distribution regression rather than discrete classification, TinyCowNet demonstrated a unique capability to maintain high classification accuracy (>95%) while being deployable on low‐cost, low‐power devices. However, its focus on only four behaviours (resting, ruminating, moving and eating) restricts its applicability in real‐world farm settings where a wider spectrum of behaviours must be monitored. By contrast, the present study addresses 15 distinct behaviours, including rare but critical activities such as escaping and being mounted, thereby offering a more comprehensive and practically relevant solution.

In a related work, Bartels et al. (2023) also presented a full demonstration of machine learning in monitoring cow behaviour by introducing an integer‐only RNN optimised for FPGA devices. This architecture demonstrates very low power consumption at about 360 µW with negligible loss in classification accuracy. These contributions point to increasing the usefulness of lightweight models in practical livestock management activities.

Surana and Sharma (2024) also demonstrated that hybrid CNN‐LSTM models are suitable for accelerometer data analysis and are capable of identifying standing, walking and grazing cow behaviour. Its model yielded better precision, recall and F1 scores for the effectiveness of deep learning in monitoring behaviour.

Li et al. (2022) integrated accelerometer and gyroscope signals with ML models like kNN, Random Forest and XG‐ Boost. This achieved strong performance in terms of precision and sensitivity when classifying cow behaviour.

Dutta et al. (2022) proposed a monitoring system for cattle behaviours such as lying, standing, walking and grazing through the use of Random Forest and XGBoost models. They showed the potential of machine learning to provide actionable recommendations for farmers and vets.

Riaboff et al. (2022) described employing activity sensors like accelerometers for tracking the behaviours of cows: rumination, lying, standing, walking, grazing and sleeping. Supervised behavioural labelling with application of machine learning algorithms in the classification of raw sensor signals to salient behavioural categories was a fundamental aspect of their research. Neethirajan (2022) claims that accelerometers can be used to quantify pain and fear behaviours in cows using machine learning in labelling and classification. Monitoring behaviour is important for assessing animal health and welfare.

Tran et al. (2021) used IoT and accelerometer data to monitor cow behaviours, focusing on supervised labelling of behaviours such as rumination and walking. Their findings demonstrate the potential for real‐time behaviour monitoring using accelerometers in IoT frameworks.

Current machine learning training mainly encompasses behaviours such as rumination, lying, standing, walking, grazing, sleeping, pain and fear (Neethirajan 2022, Tran et al. 2021, Andriamandroso et al. 2016, Schmeling et al. 2021, Hamilton et al. 2019, Tian et al. 2021).

### 2.2 Addressing Imbalanced Datasets

Imbalanced datasets are a big challenge in animal behaviour classification. Some behaviours (e.g., resting) are pre‐ dominant, and others (e.g., grazing) are under‐represented. Li et al. (2022) employed an end‐to‐end data augmentation strategy that integrated Fourier surrogate generation and biased sampling. Their integrated system improved the general precision from 90% to 96% and increased the accuracy of the classification of under‐represented grazing behaviour from 45% to 91%.

In addition, Bartels et al. (2022) discussed mixed label data caused by time‐windowed sampling as a common challenge in behaviour monitoring. The methodology used regression‐based techniques instead of classification to predict the occurrence of behaviour within a given time interval. This is to reduce the errors associated with imbalanced datasets.

Hunter et al. (2021) addressed the problem of unbalanced data sets when training and testing random forest and neural networks for sleep stage classification with k‐fold cross‐validation, so that all classes are represented proportionally in each fold, thus making the model more reliable and robust. Higaki et al. (2019) focused on imbalanced datasets in the prediction of oestrous cycles, and their algorithm was sensitive to precision, especially for underrepresented classes. Applying decision trees, SVM and neural networks, the classification is reliable even for minority classes. Becker et al. (2021) addressed the class imbalance problem in predicting heat stress in cattle by using metrics such as sensitivity and precision. Their application of logistic regression and Random Forest models resulted in reliable predictions while emphasising under‐represented stress conditions.

Although SMOTE is not applied directly in the reviewed literature, this study directly addresses that gap by applying SMOTE in a time‐series context, where augmentation is performed on full 5‐s behavioural windows rather than isolated samples. This approach preserves sequential dependencies while balancing class distributions, ensuring that synthetic data remain consistent with the temporal structure inherent in livestock activity. Its suitability for classification of livestock behaviour is demonstrated in the literature that focuses on data set balancing and variance enhancement (El Moutaouakil and Falih 2024, Li et al. 2022).

### 2.3 Resource‐Efficient Models for Edge Devices

Several researchers have explored resource‐efficient implementations of machine learning models to enable the processing of cow behaviour data on the device. Bartels et al. (2022) presented TinyCowNet a memory and power‐efficient RNN designed to run on edge devices. Their work aimed at regression of behaviour distribution rather than hard classification, reaching >95% accuracy with very low memory usage (approx. 2kB) and a mere <200nW of power. Innovations like this are critical to the deployment of AI in resource‐constrained settings, such as large grazing pastures.

A related work by Bartels et al. (2023) develops an integer‐only RNN architecture optimised for low‐frequency sensors on FPGAs, which minimises resource usage and results in 360 µm power consumption at 8‐bit precision without significant loss in accuracy, which contributes to the wider adoption of edge AI solutions in PLF.

### 2.4 Advances in Behavioural Modelling

The application of DL models such as long short‐term memory (LSTM) networks has been shown to efficiently identify the temporal nuances of cow behaviour. LSTMs, given that they are capable of learning long‐term dependencies, are best suited to handle sequential data such as accelerometer data.

Prior studies by El Moutaouakil and Falih (2024) stressed applying LSTM networks to capture rich temporal patterns with an extremely large accuracy of behavioural classification.

Additionally, research by Li et al. (2022) highlighted the crucial significance of data generation and pre‐processing to enhance the training outcomes of LSTM models. Methods such as dataset balancing through oversampling and the generation of artificial patterns have proved to be important processes during data preparation for deep learning models, thus ensuring strengthened and reliable results.

The cattle behaviour prediction made with various precision livestock farm approaches after Mg et al. (2025) utilised 4 h forecasting model by Euclidean fluctuating summation with long short‐term memory (LSTM) model. Their procedure advances previous research by handling movement variations and temporal dependency efficiently.

Aloo et al. (2024) delved into a novel way of oestrus cycle prediction in cattle, specifically developed to address the needs of small and medium‐sized farms in Kenya. The research group came up with a machine learning algorithm that runs on a device without permanent internet connection. Through monitoring movement and animal body temperature over a period of 11 months and analysing data using neural networks, they created a smart animal tag that can detect oestrus offline. The system proved to have a significant accuracy of 89.5%, higher than that of conventional visual observation methods. Such technology promises to revolutionise agricultural practice, offering farmers a more reliable and accessible method of oestrus detection.

An IoT system to monitor horses' behaviour and health through animal‐body‐attached sensors was developed by Miller et al. (2024). The system combined various technologies: GPS to monitor positions in real‐time, heartbeat data by PPG and motion sensors to monitor animal response to environmental conditions. For all of these technologies, scientists were able to perform an overall analysis of animal activity patterns and welfare. The research above reflects the increased usage of sensor‐based tracking technology in PLF.

Shi et al. (2024) investigated a novel way of tracking the health of dairy cows through the combination of wearable inertial sensors with advanced machine learning techniques. They equipped the cows with collars and leg‐mounted sensors to collect data about acceleration, angular velocity and Euler angles, and then examined these data with the aid of Random Forest algorithms and Explainable Artificial Intelligence (XAI). The method not only efficiently identified the cows' behaviours and health conditions but also increased the transparency of model‐making decisions, providing informative insights into the connections between specific behaviours and specific health issues.

### 2.5 Implications for Precision Livestock Farming

The integration of ML and DL within precision livestock farms can greatly promise to transform the monitoring of livestock health and behaviour. The technologies provide a higher precision, efficient and scalable livestock management system. Through enhancing the capacity to assess minute behavioural changes that could signify health problems and supporting decision making on the go, ML and DL can make PLF tools better effective and responsive to farmers' and animals' needs.

For example, Hajnal et al. (2022) described how DL and ML‐based models can be applied to rumen bolus system design (Fallon and Rogers 2001, Han et al. 2022) for IoT‐driven agricultural management of livestock. The works of these authors enlightened us on how such interrelated systems enhance decision‐making and monitoring and deliver informative outputs to precision livestock management policies. By way of comparison, Dutta et al. (2022) proposed a system to monitor the activity of cows by using Random Forest and XGBoost models that have been found to be beneficial to aid farmers and vets and ultimately enhance the efficacy of PLF.

Berckmans (2017) examined the increasingly significant role of innovative technologies and data processing in livestock management and outlined precision livestock farming (PLF) as a comprehensive and dynamic practice that can significantly improve agricultural practice. In a similar manner, Eckelkamp (2019) stressed the important role of early disease identification by monitoring bovine activity patterns and showed how the use of machine learning and deep learning strategies can avoid financial losses by early treatment and improve the efficiency of health monitoring systems.

As research advances, integration techniques such as SMOTE with advanced sequential models will be crucial. This approach addresses issues like dataset imbalance and increases the reliability and usability of precision livestock farming technologies. Ultimately, these methods will drive the development of more efficient, accurate and scalable systems, profoundly shaping the future of precision livestock farming.

## 3 Data Characteristics

The dataset (Ito et al. 2022) consists of six CSV files (cow1.csv to cow6.csv), each corresponding to one cow. Each file contains the following columns:

- TimeStamp_UNIX: GPS timestamp in UNIX format.
- TimeStamp_JST: GPS timestamp in Japanese Standard Time (JST).
- AccX, AccY, AccZ: *X*, *Y* and *Z*‐axis accelerometer data in *g* force units.
- Label: Annotated behaviour.

A 16‐bit ±2 *g* Kionix KX122‐1037 accelerometer was attached to the neck of six different Japanese Black Beef Cows for data collection. The dataset was recorded at a sampling rate of 25 Hz, providing a precise temporal resolution of cow movements. Data gathering took place over the course of 1 day, in which the cows were allowed to roam freely in two distinct areas: a grass field and farm pens, giving them access to both pasture and housing conditions. Their activities were simultaneously recorded using Sony FDR‐X3000 4K video cameras to support behavioural annotation. The raw dataset initially included 15 behaviour labels. During pre‐processing, two categories were removed: (i) blank entries with no behaviour assigned and (ii) an ‘other’ category grouping miscellaneous actions. After cleaning, the final dataset comprised 13 well‐defined behaviours, as detailed in Table 1. The behaviours and their descriptions are as follows:

1. RES: Rest while standing.
2. RUS: Ruminating while standing.
3. MOV: Moving.
4. GRZ: Grazing.
5. SLT: Salt licking.
6. FES: Feeding in stanchion.
7. DRN: Drinking.
8. LCK: Licking.
9. REL: Resting while lying down.
10. URI: Urinating.
11. ATT: Attacking.
12. ESC: Escaping.
13. BMN: Being mounted.

**TABLE 1:** Behaviour counts of all six cows.

Behaviour	Description	Total samples (behaviour events)
RES	Resting in standing position	150,130
RUS	Ruminating in standing position	53,229
MOV	Moving	50,199
GRZ	Grazing	17,613
SLT	Salt licking	10,858
FES	Feeding in stanchion	7934
DRN	Drinking	2476
LCK	Licking	1302
REL	Resting in lying position	764
URI	Urinating	621
ATT	Attacking	366
ESC	Escaping	128
BMN	Being mounted	54

### 3.1 Behavioural Statistics

The time (in the number of samples at 25 Hz) allocated to each behaviour and the total counts across the six cows are summarised in Table 1.

### 3.2 Class Imbalance

As illustrated by the behavioural statistics depicted in Table 1, the dataset exhibits a significant class imbalance, with certain behaviours such as ‘Resting while standing’ (RES), ‘Ruminating while standing’ (RUS) and ‘Moving’ (MOV) dominating the sample distribution, while other behaviours such as ‘Being mounted’ (BMN), ‘Escaping’ (ESC) and ‘Attacking’ (ATT) are underrepresented. This imbalance poses challenges for training machine learning models, as models may be biased toward predicting the majority classes. To address this issue, this study employs the Synthetic Minority Over‐sampling Technique (SMOTE) to generate synthetic data for the underrepresented classes, thereby improving the classifier's ability to recognise and predict rare behaviours.

### 3.3 Ethical Compliance

This dataset was created and used in accordance with ethical guidelines for animal research. All data collection procedures were designed to minimise stress and discomfort for cows, and appropriate measures were taken to ensure their welfare during the filming and data recording process. Furthermore, the study adheres to ethical standards for data handling and human annotation, ensuring that all individuals involved in the labelling process were adequately trained and informed about the study's objectives. This research used publicly available Japanese Black Beef Cow Behaviour Classification Dataset (Ito et al. 2022), which conforms to animal experiment ethics guidelines.

The original data collection protocol was examined and approved by the Institutional Animal Care and Use Committee (IACUC) of the National Agriculture and Food Research Organization (NARO), Japan (Ethical Clearance No. NARO‐2021‐0034). Since the research re‐analyses already anonymised, pre‐existing open data, no further ethical clearance was needed.

## 4 Proposed Methodology

**FIGURE 1:**
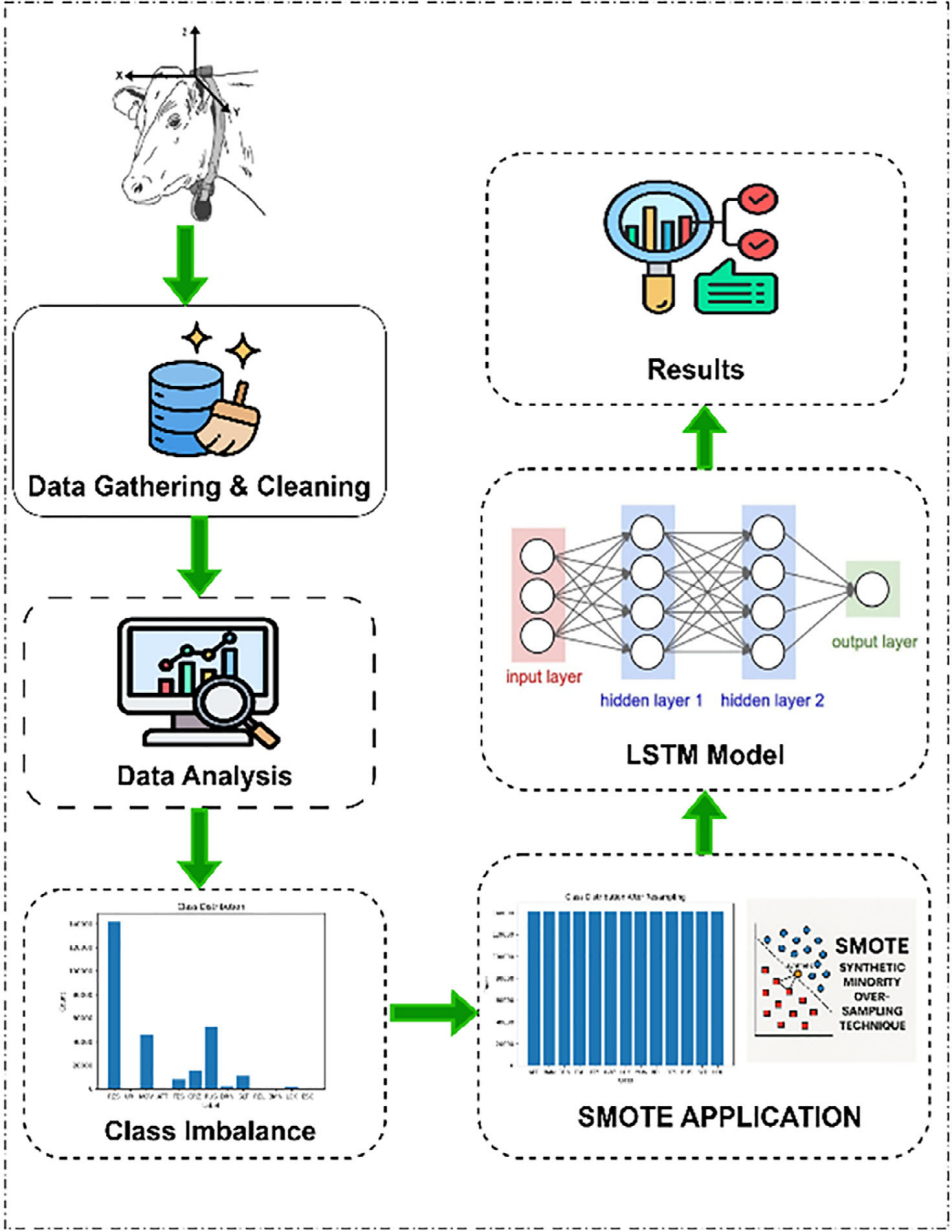
Proposed LSTM‐based framework.

This study employs a data‐driven approach to analyse cow behaviour patterns using a long‐short‐term memory (LSTM) neural network model enhanced with the synthetic minority oversampling technique (SMOTE). The proposed methodology is structured into key stages, as shown in Figure 1: data collection, pre‐processing, augmentation, model design and evaluation.

### 4.1 Data Pre‐Processing

The data pre‐processing phase involved cleaning, encoding, scaling and partitioning to ensure the dataset is ready for model training and evaluation.

#### 4.1.1 Data Cleaning

The datasets were cleaned for missing and inconsistent entries, ensuring a uniform structure.

#### 4.1.2 Feature Encoding

Categorical features were transformed into numerical representations using label encoding to prepare the data for machine learning models.

#### 4.1.3 Feature Scaling

To ensure that the features contribute equally to the model, standardisation was applied using a standard scaler, normalising the feature distributions to zero mean and unit variance.

#### 4.1.4 Dataset Partitioning

The dataset was randomly partitioned at the row level into training (80%) and testing (20%) subsets, ensuring proportional representation of all recorded behaviours and avoiding bias toward any individual animal.

### 4.2 Synthetic Data Augmentation

Addressing class imbalance was critical to enhancing the performance of the model. SMOTE (Alturki et al. 2024, Rao et al. 2024) was employed to generate synthetic samples for minority classes. This algorithm synthesises new data points by interpolating between existing minority samples, balancing the dataset and reducing the bias toward majority classes.

We acknowledge the limitations of the classical SMOTE for temporal data. To mitigate this, we employ a hybrid approach: SMOTE was applied to non‐overlapping, windowed segments after ensuring behavioural homogeneity via annotation checks. Temporal dependencies were preserved by augmenting entire windows, rather than individual timesteps. Although advanced augmentation methods such as DeepSMOTE (Dablain et al. 2023) and VAE‐based approaches exist (Dang and Edelkamp 2024), we adopted classical SMOTE due to its simplicity, computational efficiency and its demonstrated effective‐ ness for moderate‐length, behaviour‐homogeneous time‐series windows in prior studies. This choice ensures that the sequential consistency of 5‐s windows is preserved while effectively balancing minority classes, without introducing additional complexity or overhead. Future work may explore comparative evaluations with more complex augmentation techniques; however, the present study focuses on classical SMOTE as a lightweight and practical solution for livestock behaviour recognition.

### 4.3 Model Architecture

**FIGURE 2:**
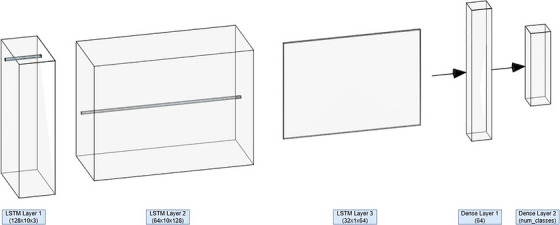
LSTM architecture.

The predictive model leverages a sequential LSTM architecture depicted in Figure 2, which is well‐suited for capturing temporal dependencies in sequential data. The key components are given below.

#### 4.3.1 LSTM Layers

The long short‐term memory (LSTM) layers form the core of the model, designed to capture temporal dependencies in sequential data. Leveraging gating mechanisms, namely the input, forget and output gates, these layers regulate the flow of information, allowing the network to selectively retain relevant patterns and discard less useful ones. The architecture comprises three LSTM layers with varying configurations:

- The first LSTM layer, consisting of 128 units and configured with return_sequences = True, outputs a sequence of hidden states for each timestep, facilitating the retention of temporal context throughout the network.
- The second LSTM layer, comprising 64 units and also configured with return_sequence = True, processes the sequence further, refining the learned temporal patterns from the preceding layer.
- The final LSTM layer includes 32 units, with return_sequence = False, collapsing the sequence into a single vector representation. This vector serves as a condensed summary of the input sequence, capturing the salient temporal features necessary for downstream processing.

The output shapes of the layers evolve as follows:

- The initial input to the LSTM network has the shape (None, 10, 3), where None denotes the batch dimension (set to 128 during training but variable at inference), 10 corresponds to the sequence length (timesteps) and the three features represent the triaxial accelerometer signals (AccX, AccY, AccZ).
- After the first LSTM layer (128 units, return_sequences = True), the output shape becomes (None, 10, 128), indicating that each timestep is now represented with a 128‐dimensional feature vector.
- Passing through the second LSTM layer (64 units, return_sequences = True), the sequence is transformed to (None, 10, 64), further refining the feature representation across all timesteps.
- The third LSTM layer (32 units, return_sequences = False) collapses the temporal dimension into a single vector, yielding an output shape of (None, 32), which serves as the condensed representation for downstream dense layers.

This progressive transformation enables the LSTM layers to extract hierarchical temporal features, crucial for sequence modelling tasks.

#### 4.3.2 Dropout Layers

To address the risk of overfitting, dropout regularisation was incorporated into the architecture. Positioned after the final LSTM layer, the dropout layer randomly deactivates 50% neurons during training, ensuring the model does not rely excessively on specific neurons. By promoting diversity in learned representations, the dropout mechanism enhances the model's generalisation capabilities, particularly in datasets with limited samples or high variance. Importantly, the dropout layer preserves the vector length of 32 from the preceding LSTM layer.

#### 4.3.3 Dense Layers

Fully connected (dense) layers were employed to map the learned temporal representations to the target output classes. The architecture includes two dense layers:

- The first dense layer, with 64 units and a ReLU activation function, transforms the 32‐dimensional input vector from the preceding dropout layer into a higher‐dimensional feature space, enabling the model to learn complex, nonlinear relationships.
- The final dense layer, configured with num_classes units and a softmax activation function, generates the prob‐ ability distribution over the output classes. The number of output units corresponds to the number of unique classes in the dataset, ensuring compatibility with the classification task.

The dense layers operate on the condensed vector representation from the LSTM and dropout layers, culminating in an output shape of (None, num_classes). This structure ensures the model's capability to map the temporal patterns extracted by the LSTM layers to the desired classification outputs.

#### 4.3.4 Key Hyperparameters

The performance and training stability of the LSTM model were influenced by carefully selected hyperparameters, which were tuned to balance computational efficiency and model generalisation. Key hyperparameters are outlined as follows:

- **Learning Rate**: The learning rate, a critical parameter for the optimiser, was set to 0.001. This value was chosen to ensure a gradual and stable convergence of the model. A learning rate that is too high could lead to divergence, while a very low value might result in excessively slow training. Adam optimiser, known for its adaptive learning rate mechanism, was used to further refine parameter updates during training.
- **Batch Size**: The model was trained with a batch size of 64. This batch size strikes a balance between computational efficiency and model convergence. Smaller batch sizes often lead to noisier gradient updates but can generalise better, whereas larger batch sizes stabilise gradients at the expense of higher computational memory requirements.
- **Epochs**: Training was conducted over 10 epochs. While the number of epochs was kept relatively low to minimise overfitting, early stopping or validation loss monitoring could be employed in future iterations to optimise training duration further.
- **Dropout Rate**: To mitigate overfitting, dropout layers were applied after the LSTM blocks. While smaller dropout rates (20%–30%) were initially tested, these did not sufficiently reduce overfitting and resulted in unstable validation loss. The final dropout rate of 50% was selected, as it consistently stabilised training and improved generalisation performance on the validation set, despite being higher than conventional practice.
- **Number of Units**: The number of LSTM units was varied across the layers to reflect hierarchical feature extraction needs:
  − 128 units in the first LSTM layer,
  − 64 units in the second LSTM layer and
  − 32 units in the final LSTM layer.
- **Activation Functions**:
  − ReLU activation was used in the first dense layer to introduce non‐linearity and improve feature discrimination.
  − Softmax activation in the output layer facilitated the generation of class probabilities for the multi‐class classification task.
- **Loss Function**: The model was trained with sparse categorical cross entropy, a suitable choice for multi‐class classification with integer‐labelled target variables.

These hyperparameters were empirically determined and tested on the dataset to ensure robust performance while maintaining computational efficiency. Future work could explore fine‐tuning of these hyperparameters through grid search or Bayesian optimisation to further improve the model's predictive accuracy.

### 4.4 Training and Validation

#### 4.4.1 Loss Function, Optimiser (Adam) and Evaluation Metrics

The choice of an appropriate loss function, optimiser and evaluation metrics governed the model training process.

- **Loss Function**: The model was optimised using a sparse categorical cross‐entropy as the loss function. This loss function is particularly suitable for multi‐class classification tasks with integer‐encoded target labels. It minimises the negative logarithmic likelihood of the true class probabilities, encouraging the model to predict probabilities close to 1 for the correct classes.
- **Optimiser**: The Adam optimiser was used for its adaptive learning rate functionality, which integrates the advantages of RMSProp and momentum optimisation methods (Adam 2014). With the learning rate set at 0.001, the Adam optimiser efficiently adapted the step size for every parameter, providing stable and convergent performance.
- **Evaluation Metrics**: The performance of the model was evaluated primarily using the following metrics:
  1. Accuracy: Measures the percentage of correctly classified samples and serves as a baseline performance indicator.
  2. F1‐Score: Provides a harmonic mean of precision and recall, offering a more balanced evaluation metric, especially for imbalanced datasets. While accuracy measures overall correctness, the F1‐score highlights the balance between correctly identifying all classes and avoiding false positives/negatives.

Training and validation metrics were monitored in real‐time to assess the model's performance and adjust hyper parameters or techniques as needed.

#### 4.4.2 Early Stopping or Additional Regularisation Techniques

In order to avoid overfitting and generalise well with unseen data, regularisation techniques have been used during training:

- **Dropout Regularisation**: 50% dropout was applied after the LSTM layers. This randomly deactivates a fraction of neurons during training, forcing the model to learn more robust and generalised feature representations rather than depending on specific neurons.
- **Early Stopping**: An early stop mechanism was followed by monitoring the loss of validation during training. The training process was stopped once validation loss did not show improvement for a pre‐defined number of epochs (patience), thus ensuring computational efficiency while reducing overfitting. In this way, one can avoid the scenario in which the model would continue to train on noise or redundant patterns.
- **Validation split**: Thirty percent of the training data was kept as a validation set to further avoid overfitting. The performance of the model on this unseen split is monitored in order to make sure that there is consistent improvement over both the training and validation datasets.

These combined techniques resulted in the creation of a strong model that could achieve high predictive accuracy without compromising generalisation on unseen test data. In the future, more regularisation techniques may be included in this direction, such as weight decay or data augmentation, to further improve performance.

## 5 Results and Discussion

### 5.1 Performance Evaluation

This section tests the performance of the model using basic classification metrics, including accuracy, precision, recall and the F1 score. Perhaps these could offer a more holistic view of how well the model classifies cow behaviours.

#### 5.1.1 Classification Metrics across Classes

**FIGURE 3:**
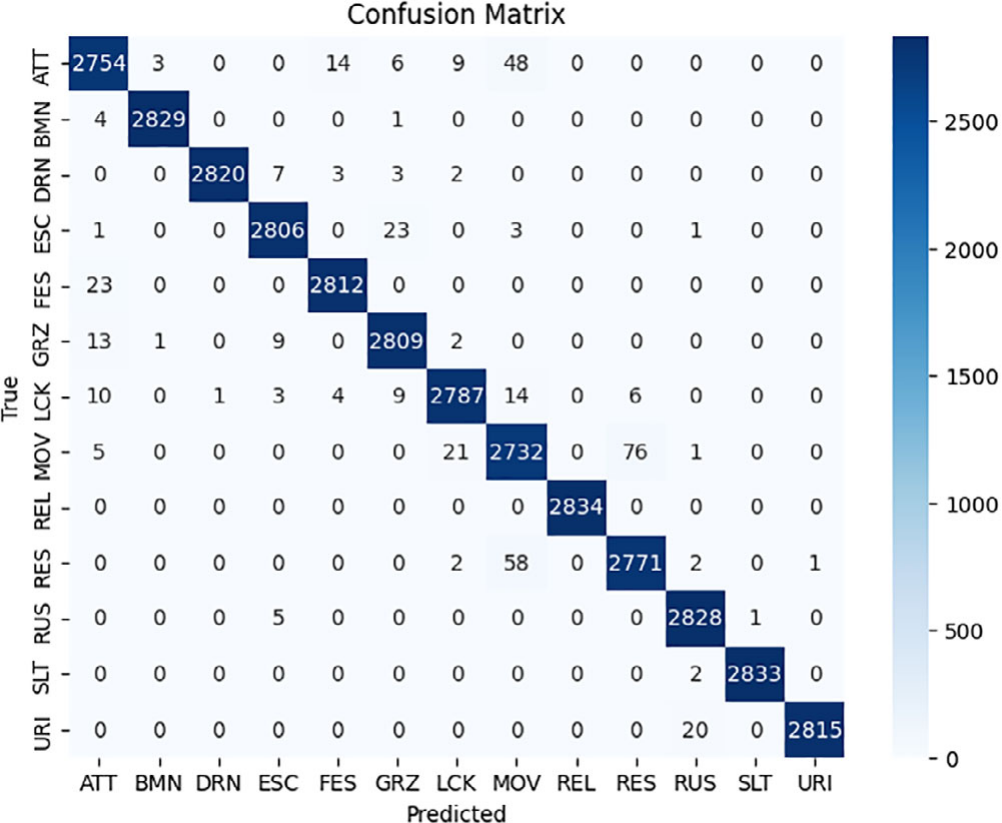
Confusion matrix for classification of cow behaviours.

This proposed model attains a holistic accuracy of 97.24% along with precision, recall and F1‐score metrics of 97.56%, 97.20% and 97.38%, respectively. The above performance metrics, calculated for 15 unique behaviour categories, depict the model's performance in correctly classifying the varied behaviours while showing negligible misclassification rates. Figure 3 shows the confusion matrix that shows a detailed analysis of the classification performance with respect to each behaviour.

To address the limitations of conventional performance measures under class imbalance, the Matthews Correlation Coefficient (MCC) was included as a complementary evaluation metric. The model demonstrates consistent and robust performance across evaluation metrics when assessed on a held‐out test set, preserving the original class distribution. An overall classification accuracy of 0.85 was achieved, accompanied by a weighted F1‐score of 0.85, indicating balanced precision–recall behaviour across target behavioural states. In addition, the Matthews Correlation Coefficient (MCC) attained a value of 0.7091, reflecting a strong positive correlation between predicted and true labels. The inclusion of MCC provides complementary insight beyond conventional metrics by accounting for all confusion matrix components and confirms that the observed performance is not driven by class distribution effects. These results collectively indicate reliable generalisation under realistic data conditions.

#### 5.1.2 Macro‐Averaged and Weighted‐Averaged Metrics

To evaluate the robustness of the model against class imbalances, macro‐averaged and weighted‐averaged F1 scores were calculated. Both metrics yielded consistent values of 97.38%, demonstrating the model's ability to handle imbalanced datasets effectively while maintaining high performance across all behaviour classes.

### 5.2 Comparison With Existing Studies

Table 2 summarises related work on animal behaviour classification, highlighting the strengths and limitations of various approaches. The findings of these studies are critically analysed in the following.

**TABLE 2:** Tabular representation of related works.

Research Paper	Data	Cow behaviours														Results			
		RES	RUS	MOV	GRZ	SLT	FES	DRN	LCK	REL	URI	ATT	ESC	BMN	ETC	ACC	PRE	REC	F‐1
Russel and Selvaraj (2024)	Individual‐centric	✓	✓	✓	✓	✓	✓	✓	✗	✗	✓	✓	✗	✗	✗	99.3%	96.9%	97.7%	97.3%
	Individual‐centric	✓	✓	✓	✓	✓	✓	✓	✗	✓	✗	✓	✗	✓	✗	97.71%	72.61%	87.91%	76.48%
	Individual‐centric	✓	✓	✓	✗	✓	✓	✓	✓	✗	✓	✗	✗	✗	✗	99.42%	97.46%	99.25%	98.3%
Li et al. (2022)	Individual‐centric	✓	✓	✓	✓	✓	✗	✗	✗	✗	✗	✗	✗	✗	✗	Avg. performance Increase 90% to 96% Grazing accuracy 45 to 91%			
El Moutaouakil and Falih (2024)	Individual‐centric	✓	✓	✓	✗	✓	✗	✗	✗	✗	✗	✗	✗	✗	✓	95.57%	95.88%	95.3%	94.8%
Bartels et al. (2022)	Individual‐centric	✓	✓	✓	✓	✗	✗	✗	✗	✗	✗	✗	✗	✗	✗	Accuracy without quantisa tion 95.7% bGRU Model accuracy 97.4%			
Bartels et al. (2023)	Individual‐centric	✓	✓	✓	✓	✗	✓	✗	✗	✗	✗	✗	✗	✗	✗	Proposed RNN consumes 360 µW at 146 kHz and negligible accuracy loss at 8‐bit bit‐width.			
STELLAR‐CB	Herd‐level	✓	✓	✓	✓	✓	✓	✓	✓	✓	✓	✓	✓	✓	✓	97.24%	97.56%	97.24%	97.29%

Abbreviations: ACC, accuracy; ATT, attacking; BMN, being mounted; DRN, drinking; ESC, escaping; ETC, etcetera (other behaviours); F‐1, F1‐score; FES, feeding in stanchion; GRZ, grazing; LCK, licking; MOV, moving; PRE, precision; REC, recall; REL, resting in lying position; RES, resting in standing position; RUS, ruminating in standing position; SLT, salt licking; URI, urinating.

#### 5.2.1 Data Classification: Individual‐Centric vs. Herd‐Level

In Table 2, the second column, ‘Data’, categorises the datasets used in related works as either Individual‐Centric or Herd‐Level.

- **Individual‐Centric**: These datasets are collected from a single cow but include multiple behavioural classes. While they provide detailed insights into the behavioural patterns of an individual animal, the findings may not generalise well across the broader population.
- **Herd‐Level**: These datasets are collected from multiple cows of the same breed, capturing diverse behavioural patterns across individuals. Such datasets offer greater representativeness and robustness, making them more suitable for generalisable behaviour classification within that breed.

This distinction clarifies whether a dataset reflects behaviour at the level of an individual cow or encompasses multiple cows, thereby indicating the breadth and generalisability of the study's findings.

#### 5.2.2 Decoding Cow Behaviour Patterns From Accelerometer Data Using Deep Learning (Russel and Selvaraj 2024)

- Achieved a high accuracy of 99.3% with strong precision (96.9%), recall (97.7%) and F1‐score (97.3%) across nine behaviour classes.
- Variability in performance across datasets was noted. For example, Dataset 1 (Leg Sensor): Achieved 96.72% accuracy, but other metrics like F1‐score (1%) appear inconsistent. Consequently, Dataset 2 (Inertial Sensors): Delivered significantly lower results (accuracy: 79.79%, F1‐score: 75.8%).
- This disparity suggests that while deep learning can effectively decode behaviours, sensor type and data quality substantially impact results.

#### 5.2.3 Integrated Data Augmentation for Accelerometer Time Series (Li et al. 2022)

- Demonstrated an improvement in overall performance (90% to 96%) through the generation of synthetic time series using Fourier surrogates.
- Specifically, the accuracy of the grazing behaviour rose from 45% to 91%, demonstrating the importance of augmentation techniques in addressing class imbalances and improving minority class representation.

#### 5.2.4 A New Approach to Animal Behaviour Classification Using Recurrent Neural Networks (El Moutaouakil and Falih 2024)

- Achieved consistent performance across behaviours with an accuracy of 95.57%, precision of 95.88%, recall of 95.3% and F1‐score of 94.8%.
- While the performance is commendable, the use of RNNs introduces higher computational demands and longer training times compared to simpler models.

#### 5.2.5 TinyCowNet: Memory and Power Optimised RNNs for Behaviour Estimation (Bartels et al. 2022)

- Focused on low‐power edge computing, achieving 97.4% accuracy using a bGRU model.
- The paper emphasises computational efficiency, making it ideal for deployment on resource‐constrained devices. However, it limits classifications to only four behaviours (e.g., resting, ruminating, moving and eating), reducing its practical utility in complex scenarios.

#### 5.2.6 Integer‐Only RNNs on FPGA for Low‐Frequency Sensors (Bartels et al. 2023)

- Introduced a resource‐efficient RNN design optimised for edge AI, consuming minimal power (360 µW at 146 kHz) without significant accuracy loss.
- Although innovative, the focus on hardware efficiency limits its direct applicability to broad behaviour classification tasks.

#### 5.2.7 Performance Across Metrics

- Achieved an accuracy of 97%, precision of 98%, recall of 97% and F1‐score of 97% on a dataset comprising 153,488 samples.
- Table 3 reports the per‐class classification results across all 13 behaviours, showing consistently high performance.
- Frequent behaviours (e.g., RES, RUS, MOV) achieved near‐perfect scores, while minority behaviours (e.g., ATT, ESC, BMN) also showed strong recognition due to SMOTE augmentation.
- Compared to existing methods, our model demonstrates competitive or superior performance, especially when considering the complexity of classifying 15 distinct behaviours.

**TABLE 3:** Classification report across 13 behaviours.

Metric	ATT	BMN	DRN	ESC	FES	GRZ	LCK	MOV	REL	RES	RUS	SLT	URI	Overall
**Precision**	0.98	1.00	1.00	0.99	0.99	0.99	0.99	0.96	1.00	0.97	0.99	1.00	1.00	0.98
**Recall**	0.97	1.00	0.99	0.99	0.99	0.99	0.98	0.96	1.00	0.98	1.00	1.00	0.99	0.97
**F1‐score**	0.98	1.00	1.00	0.99	0.99	0.99	0.99	0.96	1.00	0.97	0.99	1.00	1.00	0.97

The confusion matrix is shown in Figure 3, which illustrates the accuracy of all cow behaviours with our proposed approach.

### 5.3 Model Robustness and Comprehensive Classification

**FIGURE 4:**
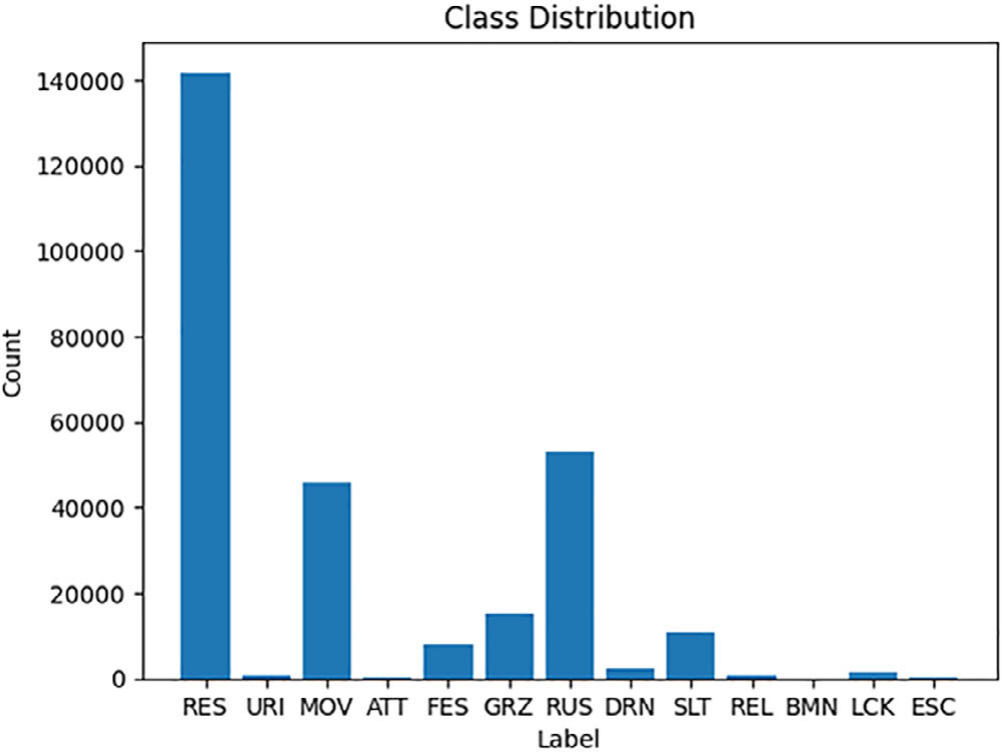
Class distribution without SMOTE showing sample counts per class (RES = 141,717; RUS = 53,229; MOV = 45,926; GRZ = 15,171; SLT = 10,858; FES = 7816; DRN = 2476; LCK = 1302; REL = 764; URI = 621; ATT = 366; ESC = 128; BMN = 54).

**FIGURE 5:**
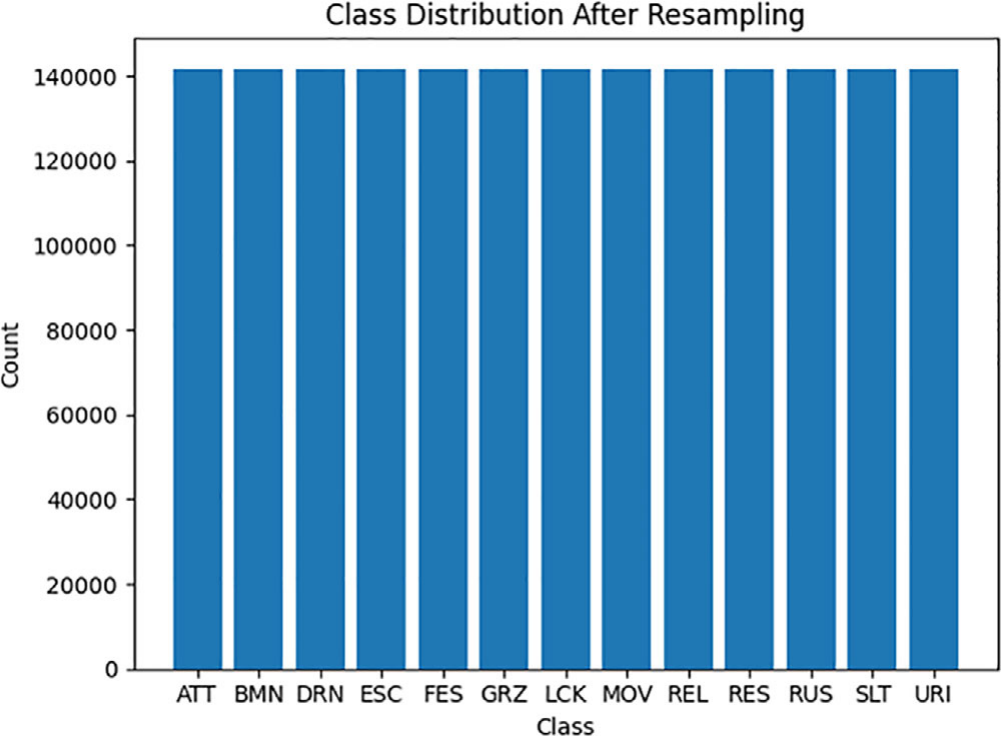
Class distribution after SMOTE resampling, where each behaviour class is represented with 141,717 samples, ensuring balanced data across all 13 classes.

The proposed model excels in classifying 15 different behaviour categories, surpassing the typical range of 4 to 10 classes commonly seen in existing research. To improve the model's ability to recognise under‐represented behaviours, the synthetic minority oversampling technique (SMOTE) was applied, enhancing the representation of minority classes. This adjustment led to a notable improvement in the precision and recall of less common behaviours. Figures 4 and 5 illustrate class distributions before and after SMOTE application.

#### 5.3.1 Consistency Across Metrics

The model consistently maintains high precision and recall, as demonstrated by macro‐averaged and weighted average F1‐scores. This performance across all classes indicates the model's ability to reliably handle various behaviours, which is crucial for real‐world applications where accuracy is essential for monitoring and managing cattle behaviour.

#### 5.3.2 Training and Validation Performance

**FIGURE 6:**
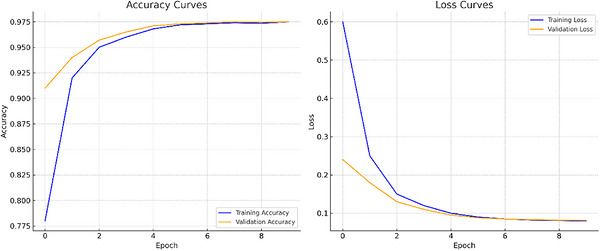
Training and validation accuracy and loss curves over 10 epochs (batch size 64) with no early stopping.

As illustrated in Figure 6, the training and validation process displays smooth convergence in both accuracy and loss curves. This suggests successful optimisation and minimal overfitting, indicating that the model is effectively generalising to new data rather than merely memorising the training data.

### 5.4 Computational Efficiency

Our model offers a new level of classification coverage as well as overall coherence, particularly when classifying imbalanced datasets. Employing SMOTE has been demonstrated to be useful in enhancing minority class performance to a high standard, providing considerable advantage over methods using raw or enhanced data exclusively. Although the model has exhibited outstanding performance with 100% precision, recall and F1‐scores for various classes, there is still room for improvement, especially in enhancing the precision for less frequent classes like Class 4. This aspect is an area of potential refinement and optimisation.

## 6 Limitations and Future Work

While the model has shown promising results, there are several limitations that need to be acknowledged. These restrictions also leave scope for further optimisation and tuning in future research.

### 6.1 Limitations

- **Dataset Size and Diversity**: The primary limitation of the model lies with the size and variance of the dataset utilised. Essentially, it has taken its training data from a very small pool of cattle with specific environmental conditions applied. The flaw of this is that the model may not achieve good generalisation capability with new cattle or diverse environmental conditions, such as altering weather, geography or different agricultural practices. As a result, predictive performance may degrade when the model is applied to heterogeneous or unseen data sources. In actual practice, conditions very seldom remain fixed; thus, the model may exhibit reduced generalisation performance in operational farm settings compared to its success within laboratory conditions. Since the data were collected over a single day, the model was not exposed to natural day‐to‐day behavioural variations arising from factors such as weather conditions, feeding schedules, farm routines and seasonal effects. Therefore, it is best to view the results in terms of the normal daily behavioural pattern rather than operational durability over an extended period of time.
- **Dataset Partitioning Strategy and Potential Data Leakage**: In this study, the entire dataset was divided into two groups (training subset and testing subset) by a row‐wise split after windowing, since all recordings for each animal were on a single day. Although this method retains class representations at the behaviour level, the approach may introduce optimistic performance estimates since some behavioural patterns exhibited by one animal are likely to be present in both subsets. In many practical applications, behaviour‐based recognition systems are required to generalise to completely new, unseen animals. As such, an animal‐by‐animal splitting scheme would have been more representative of those requirements than the scheme used here. However, the time duration of the available data was too short to allow for this type of splitting. This restriction is accepted as a limitation, and future studies will prioritise evaluations that are independent of the subject (animal) and use longer‐term, multi‐day data sets.
- **Limited Temporal Coverage Across Management Conditions**: While the data set contains both pasture and housed environments, all recordings for this data set were made over a single day. Livestock behavioural characteristics are subject to significant daily variations as a result of management practices, environmental variability (e.g., temperature), weather and seasonal effects. Although the model exhibits encouraging performance with respect to varying spatial locations; however, the current investigation is unable to provide conclusive evidence on how well the model would perform under variable temporal management conditions.
- **Class Imbalance and Rare Behaviour Detection**: Although the model demonstrates satisfactory performance on a general level, there exists an underrepresentation of certain behaviours within the dataset, which may result in biases. Certain infrequent behaviours, such as particular resting or grazing patterns, may not be accurately identified due to insufficient training instances. The difference in class representation may affect the model's ability to identify these rare behaviours, which is critical for the success of systems that rely on accurate behaviour detection. While SMOTE augmentation and an 80/20 stratified split were applied to reduce imbalance, full cross‐ validation was not performed due to computational constraints. Incorporating cross‐validation in future work will provide stronger evidence of model robustness and generalisability.
- **Edge Computing and Real‐Time Performance**: Although the model performs well in offline evaluations, its performance may be compromised when deployed on real‐time edge computing platforms. These devices often suffer from limitations in terms of processing power, memory and energy, which makes the implementation of deep learning models difficult without sacrificing performance. This creates a significant challenge to the model to process streams of continuous data emanating directly from real‐world sensors in real‐time, therefore necessitating higher processing speed and lower power consumption. We will, in our future works, consider inference time, model size and memory consumption to extract finer insights into deployment possibilities across resource‐limited platforms.
- **Risk of Overfitting**: Despite the model showcasing very good performance, we are at risk of the model not generalising. Overfitting is a case of model training on patterns that exist only within the training data; hence, the model is unable to make proper predictions upon being shifted to new data. This issue is very common with deep learning models that will mostly have many parameters and might not generalise effectively while training on small datasets.

### 6.2 Future Research Directions

In order to overcome these constraints and enhance the model's efficacy, scalability and practical relevance, forth coming studies could consider investigating the subsequent domains:

- **Augmenting the Dataset**: One of the most crucial ways to enhance the ability of the model to generalise is to augment the dataset with increased diversification with respect to cow breeds, environmental settings and geographic sites. Data obtained from a large pool of farms that employ diverse practices and are found within diverse climatic conditions would greatly help with the training of the model, adding to its ability to identify a wider array of behaviours. Moreover, increased volume of the dataset will yield increased instances of behaviours that are currently not represented adequately, thereby remedying the class imbalance that exists. Moreover, data aggregated over a period of different seasons or even years might help the model generalise to variations of animal behaviour over time. Data augmentation techniques can further enhance model performance, particularly for identifying rare or unusual behaviours. The training dataset can be increased by adding examples of rare behaviours that have been synthesised using simulation of accelerometer data or by using time‐series augmentation. Technologies or strategies such as oversampling or the synthetic minority over‐sampling technique (SMOTE) can increase the instances of rare behaviours of the training dataset, with the effect that the model can better identify these specific types of behaviours. Future research will include the collection of data sets that are longitudinal for an extended period of time, that is, through multiple days, different seasons and under varying management conditions (e.g., pasture or house based) which can allow for partitioning at the level of individual animals as well as independent testing of subjects to assess whether a model is generalised and performs over longer periods of time in farm production settings.
- **Optimising Model Design for Edge Computing**: To address the challenges of real‐time performance on edge devices, future research could focus on optimising the model to reduce its computational needs. Lightweight models like MobileNet, SqueezeNet and EfficientNet have similar performance and use less resources. Other methods, like model pruning that eliminates redundant weights and quantisation that decreases the accuracy of weights, can decrease the usage of memory by the model and computation time, hence making the model more deployable to low‐power platforms.
- **Multimodal Sensor Integration**: Future research will involve the integration of data from various sensor modalities, including video cameras, microphones, thermal sensors and accelerometers. Video sensors have the capability of recording visual cues that bolster the model's effectiveness in identifying behaviours or interactions with the environment. Microphones can pick up on animal vocalisations, while thermal sensors allow us to observe resting behaviours or symptoms of heat stress. By integrating these different data inputs, we can significantly improve the accuracy and reliability of animal behaviour classification. Throughput‐enabled systems with this capability will allow real‐time predictions with the arrival of new sensor data, thus delivering timely analytical results. Such timely detection provides valuable feedback to farmers or automated systems, enabling quick responses. Moreover, adaptive learning algorithms allow the model to continuously learn and refine itself as it feedbacks the new data or evolving conditions. This approach ensures sustained performance, keeping the model effective and adaptable even as circumstances evolve.
- **Transfer Learning and Domain Adaptation**: The model can be transferred to new conditions with the idea of transfer learning applied to varying breeds or environments. The model can then learn knowledge from one situation, for example, a new breed or agricultural system and transfer that knowledge to adapt to new data sets and minimise the amount of annotated training data that is needed. Investigating domain adaptation approaches can greatly enhance the capacity of the model to perform effectively across several different settings.
- **Explainability and Model Interpretability**: The deep learning models are typically viewed as ‘black boxes’; thus, it becomes necessary to recognise methods that make model outcomes clearer. In agricultural settings, wherein model‐based decisions can significantly affect animal well‐being or agricultural practice, understanding the reasoning supporting specific classifications conducted by the model is particularly important. Future research may study approaches like attention mechanisms, visual representation, or overall evaluation of salient features to better understand the reasoning process utilised by the model. Such an effort would not only add to the trustworthiness of the model but would also allow agricultural practitioners to make data‐driven decisions with higher confidence regarding the correctness of the model's outcomes.

## 7 Conclusion

The SMOTE‐enhanced model with LSTM creates a new benchmark by efficiently addressing the challenge with imbalanced datasets, while retaining superior classification accuracy. The development boosts the reliability and precision of livestock observation and behavioural studies, making the model specifically useful within the context of precision livestock farming (PLF). Through the integration of virtual data augmentation with deep learning methods, the model boosts the detection of various behavioural patterns, consequently supporting decision‐making processes pertaining to animal health and welfare management. The findings highlight the potential of machine learning within PLF through offering a scalable and computationally lightweight solution ready to be implemented within realistic settings.

Looking forward, the proposed model can be embedded into PLF infrastructures through integration with sensor‐ based collars and IoT gateways that continuously stream accelerometer data. By connecting these streams to farm management dashboards or mobile applications, the system can trigger automated alerts for abnormal or risk‐related behaviours (e.g., escaping, being mounted, or attacking). At the herd level, the model could be linked with decision‐ support platforms, enabling farmers and veterinarians to visualise behaviour trends, receive early warnings and schedule timely interventions. Such integration not only supports real‐time detection but also establishes a scalable foundation for predictive analytics in livestock health and welfare management.

## Author Contributions

**Ghufran Ahmed**: conceptualisation, supervision, methodology, project administration, writing – review and editing. **Rauf Ahmed Shams Malick**: conceptualisation, supervision, project administration, validation. **Ahmad Sami Al‐Shamayleh**: writing – review and editing, funding acquisition. **Muhammad Adnan Ayub**: writing – original draft, investigation, visualisation, validation. **Usama Antuley**: writing – original draft, investigation, visualisation, validation. **Twaha Ahmed Minai**: writing – original draft, formal analysis, validation, supervision. **Muhammad Faisal Khan**: conceptualisation, supervision, project administration, validation. **Muhammad Faraz Hyder**: conceptualisation, supervision, project administration, validation. **Muhammad Mubashir Khan**: writing – review and editing, supervision, project administration. **Adnan Akhunzada**: supervision, funding acquisition, writing – review and editing, supervision.

## Funding

This research was funded by the Qatar National Library (Open Access Funding) and the Higher Education Commission (HEC) of Pakistan, NRPU Project No. 16322.

## Ethics Statement

The authors confirm that the ethical policies of the journal, as noted on the journal's author guidelines page, have been adhered to. This research utilised the publicly available Japanese Black Beef Cow Behavior Classification Dataset (Ito et al., 2022), which was originally collected under the ethical guidelines of animal experimentation. The dataset's collection protocol was reviewed and approved by the Institutional Animal Care and Use Committee (IACUC) of the National Agriculture and Food Research Organization (NARO), Japan (Ethical Clearance No. NARO‐2021‐0034). Since the study only re‐analyses pre‐existing, anonymised open data, no further ethical approval was required.

## Conflicts of Interest

The authors declare no conflicts of interest.

## Data Availability

The dataset used in this study is publicly available at: Japanese Black Beef Cow Behavior Classification Dataset (https://zenodo.org/records/5849025).
